# Gastrointestinal and Hepatic Involvement in Hypereosinophilic Syndrome

**DOI:** 10.7759/cureus.760

**Published:** 2016-08-31

**Authors:** Faisal Inayat, Abu Hurairah

**Affiliations:** 1 Department of Medicine, New York-Presbyterian Hospital, Weill Cornell Medical College, New York City, NY, USA; 2 Division of Gastroenterology, Department of Medicine, SUNY Downstate Medical Center, Brooklyn, NY, USA

**Keywords:** hypereosinophilic syndrome, hepatitis, jaundice, colitis

## Abstract

The objective of this investigation was to study the gastrointestinal and hepatic involvement in hypereosinophilic syndrome (HES). Gastrointestinal or hepatic involvement is estimated to affect up to one-third of patients with HES, although most of the clinical evidence has been derived from case reports. In literature, HES presenting with hepatitis and jaundice with subsequent development of colitis is a rare clinicopathologic entity. Given the clinical implications, physicians should include HES among differentials in these types of presentations.

## Introduction

Hardy and Anderson first described the term hypereosinophilic syndrome (HES) in 1968. It is characterized by sustained eosinophilic overproduction [1]. HES is a rare disease that usually occurs in 20–50-year-old individuals. It is categorized as primary due to neoplasm, secondary or reactive and idiopathic. HES can involve any organ, and it has been noted in one retrospective multicenter study that it involves the skin in 37%, the lungs in 25%, the gastrointestinal tract in 14%, the heart in five percent, and the central nervous system in four percent of the cases [2].

Idiopathic HES was first defined by Chusid, et al. [3] as the sustained elevation of eosinophil count more than 1500 cells/mL for more than six months with single or multiorgan dysfunction due to cytotoxic injury by eosinophils and the absence of identifiable etiology of elevated eosinophils [3]. It is a diagnosis of exclusion, which needs extensive workup and requires both invasive and noninvasive investigations to establish the diagnosis.

Herein, we would like to share an interesting case of a patient with idiopathic HES who presented with hepatitis and jaundice with subsequent development of colitis. We undertook the discussion of important clinical implications associated with gastrointestinal and hepatic involvement in HES. Informed consent was obtained from the patient for this study.

## Case presentation

A 20-year-old male presented to our institution with a seven-month history of progressively worsening jaundice. The patient denied abdominal pain, nausea, and vomiting. His past medical history was unremarkable with no preexisting gastrointestinal or liver illnesses. He had not recently taken any medication. The patient was a nonsmoker, nonalcoholic, and he did not have a history of illicit substance use. The physical exam was unremarkable other than a malnourished physique with a BMI of 18.6 (normal, 18.5–24.9).

The initial laboratory studies revealed alanine transaminase 1204 IU/L (normal, 10–40 IU/L), aspartate transaminase 721 IU/L (normal, 5–40 IU/L), serum alkaline phosphatase 153 IU/L (normal, 32–92 IU/L), total bilirubin 9.7 mg/dL (normal, 0.3–1.2 mg/dL), and international normalized ratio (INR) 1.24 (normal, 0.8–1.2). The complete blood count was as follows: WBC 25,200/mm3, hemoglobin 12.6 g/dL, platelets 499,000/mm^3^, eosinophils 67%, absolute total eosinophils 16,884/mm^3^. The serum IgG level was 1620 mg/dL (normal, 700–1600 mg/dL). The patient had no evidence of allergic or hypersensitivity conditions or connective tissue diseases based on bronchoscopy, pulmonary function tests, and serologic tests. A transthoracic echocardiogram was unremarkable. A bone marrow biopsy for neoplastic or primary bone marrow disorders showed marked eosinophilia, but no other abnormalities.

A workup for infectious causes demonstrated negative results for viral markers (hepatitis A, B, C, E, adenovirus, cytomegalovirus, and human immunodeficiency virus), serum antibodies to helminth (worm) parasites, autoimmune antibodies (antinuclear, anti-dsDNA, and anti-neutrophilic cytoplasmic antibodies), tumor markers (alpha-fetoprotein, carbohydrate antigen 19-9, and carcinoembryonic antigen), and rheumatoid factor. No pathogens were detected in a microbiological blood culture, and the stool examinations for parasites and protozoa were negative. The flow cytometry was normal.

**Figure 1:**
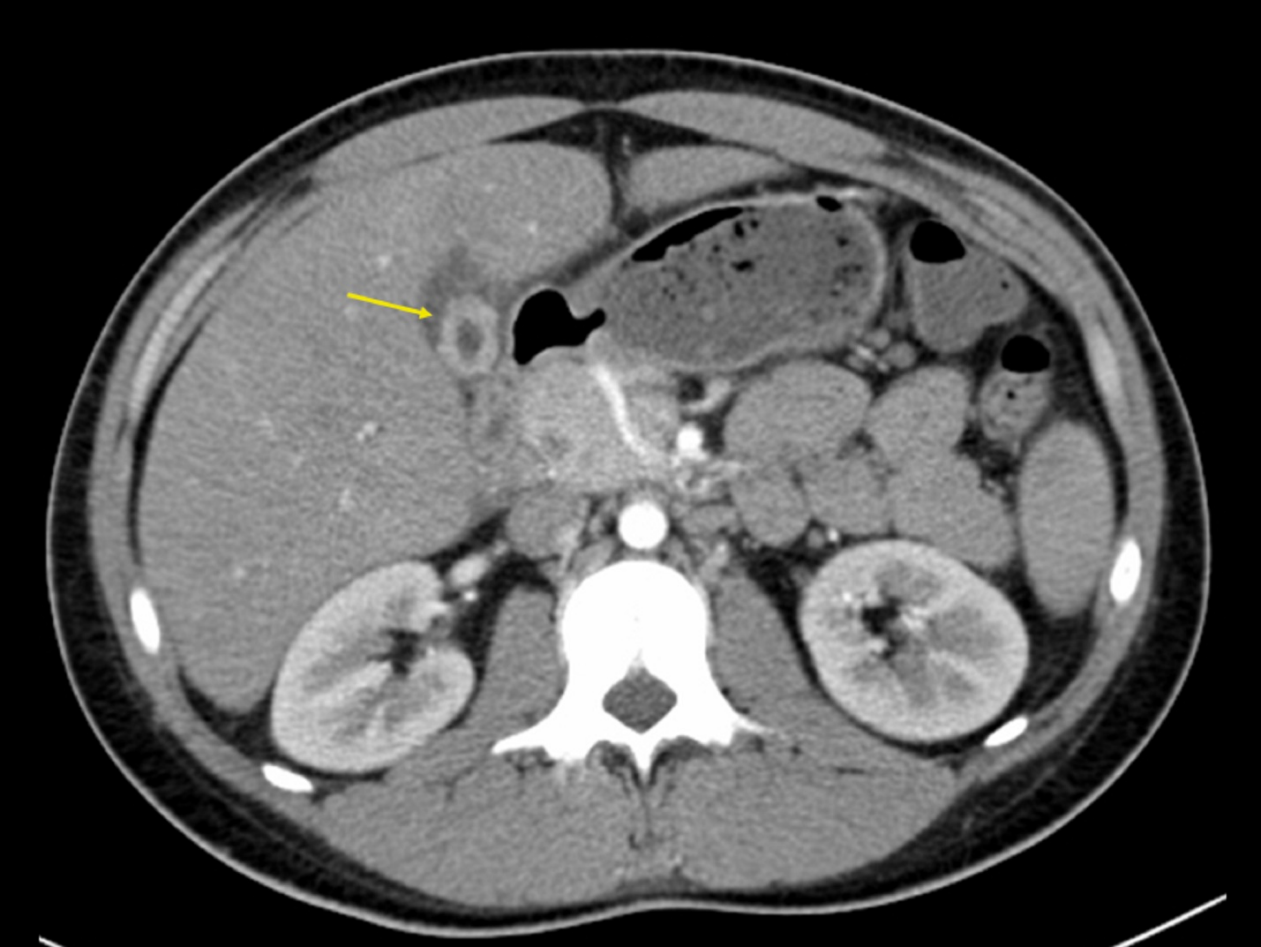
A Computed Tomography Scan of the Abdomen The arrow demarcates a thick-walled gallbladder with a rim of pericholecystic fluid present in the gallbladder fossa with a normal-appearing liver.

A computed tomography scan of the abdomen demonstrated a thick-walled gallbladder with a rim of pericholecystic fluid present in the gallbladder fossa with a normal-appearing liver (Figure 1).

**Figure 2:**
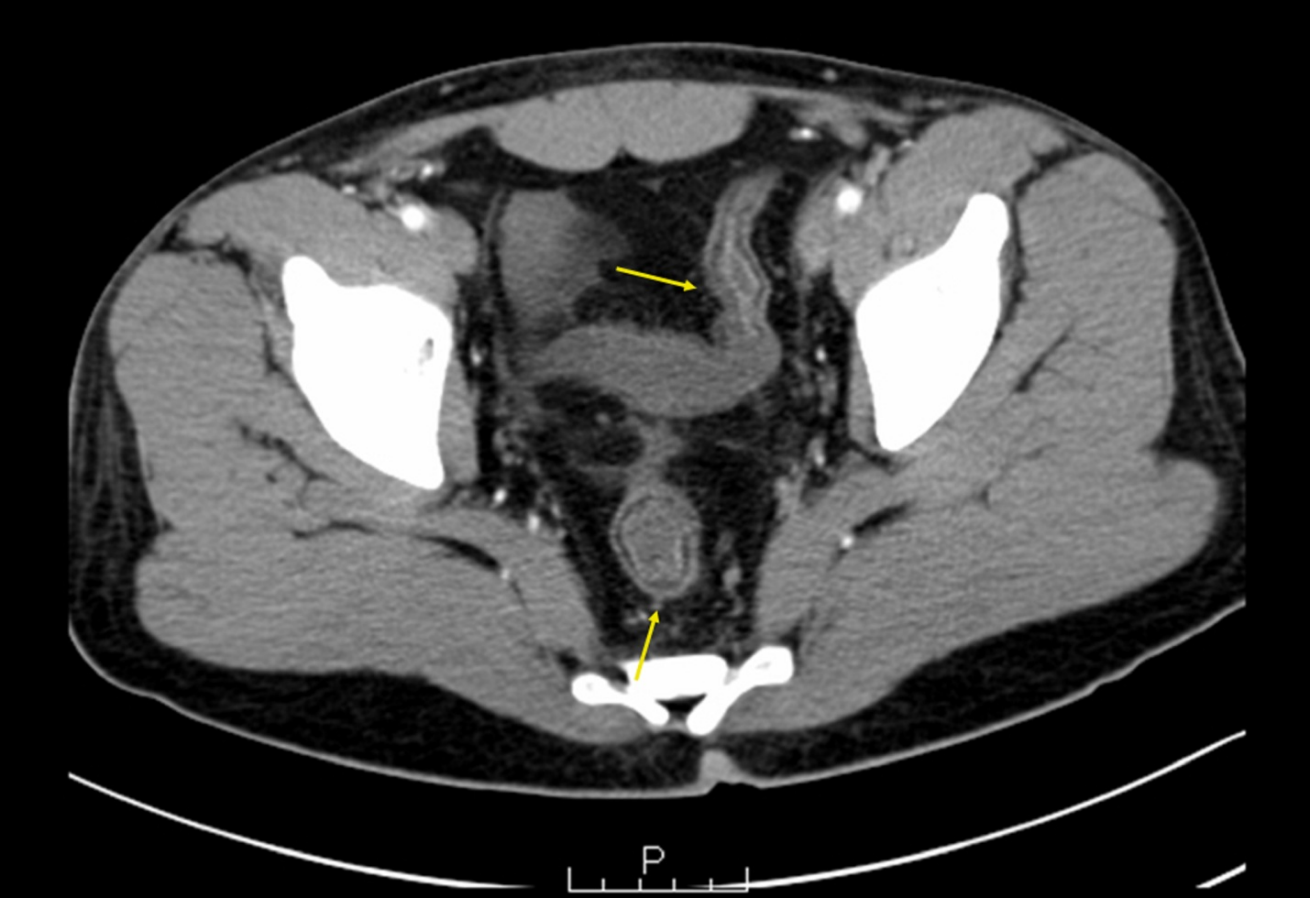
A Computed Tomography Scan of the Pelvis The arrows show loss of normal mucosal pattern with an ahaustral appearance of the colon with normal wall thickness of the rectum. The perirectal fat planes appeared to be preserved.

Furthermore, the colon showed a loss of normal mucosal pattern with no visible haustrations. The rectum had a normal wall thickness, and the perirectal fat planes appeared to be preserved (Figure 2).

**Figure 3:**
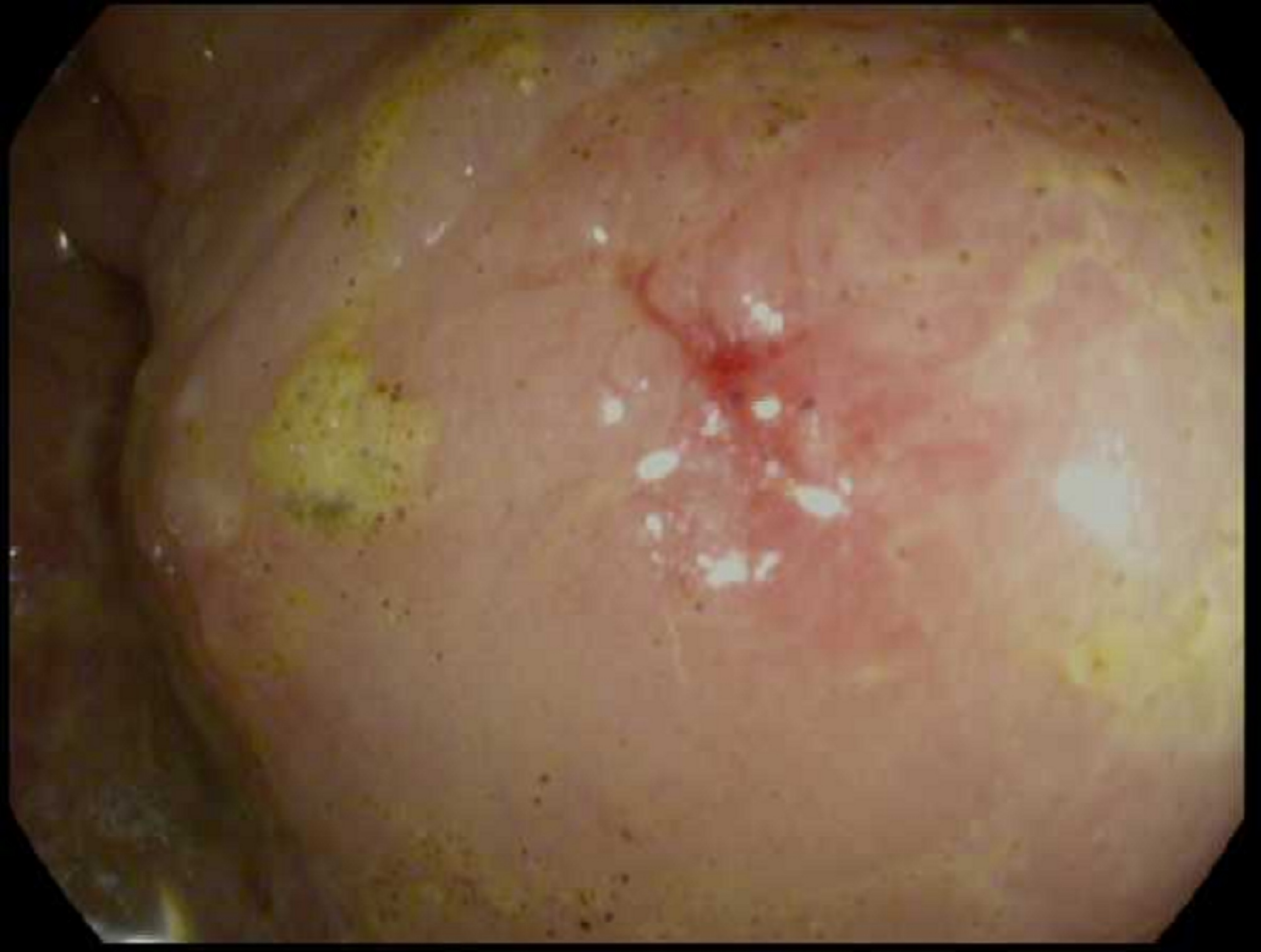
Sigmoidoscopy A colonoscopy image showing mild inflammatory changes, mucosal edema, and patchy erythema.

A flexible sigmoidoscopy exam was performed to assess colonic tissues following radiologic manifestations. It revealed mild inflammatory changes, multiple mucosal erosions, and patchy erythema (Figure 3).

**Figure 4:**
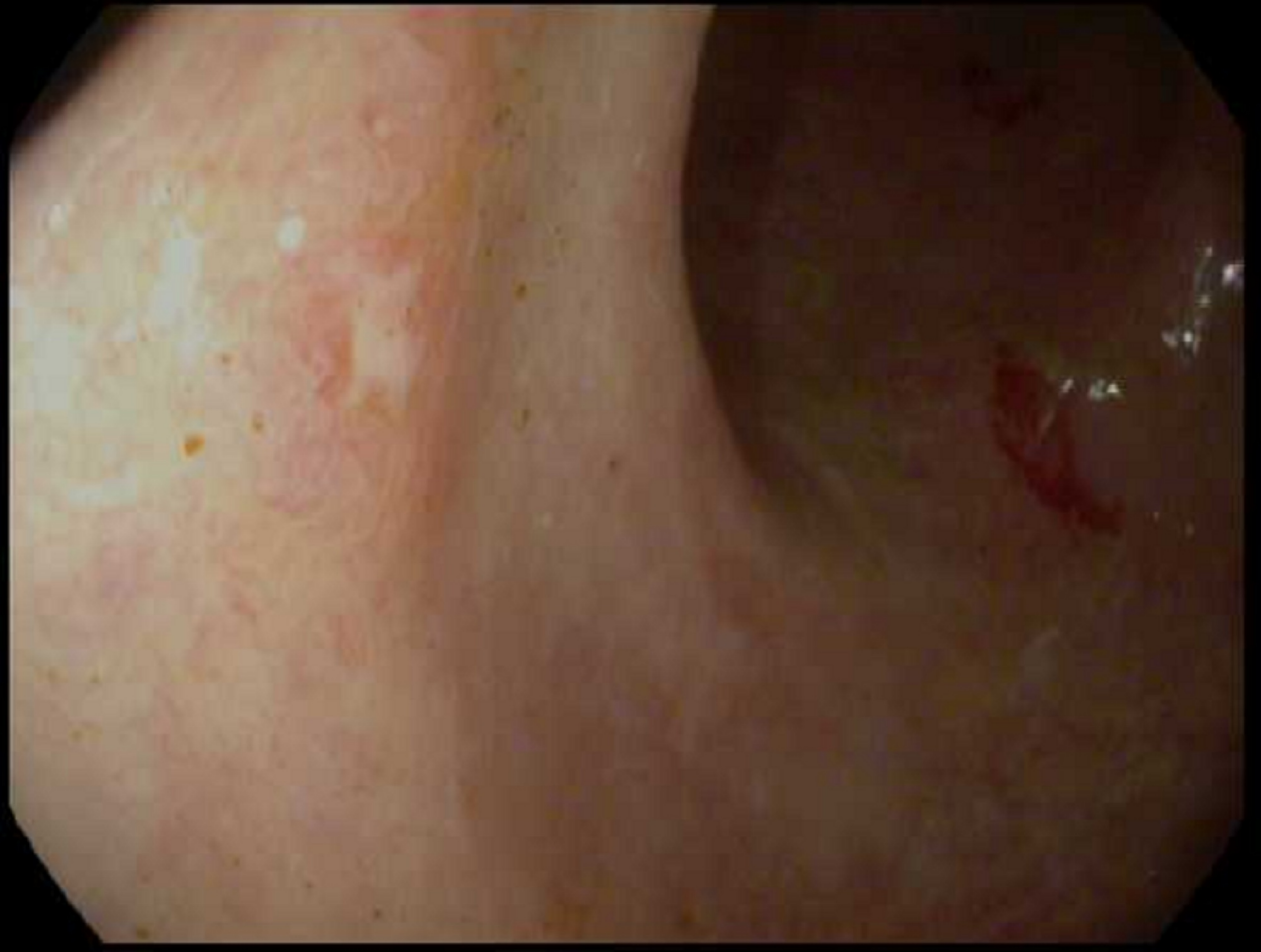
Sigmoidoscopy A colonoscopy image showing mild inflammatory changes, mucosal edema, patchy erythema, and loss of vascularity consistent with mildly-active chronic colitis.

In addition to the other findings on colonoscopy, the loss of vascularity was evident. These findings were consistent with mildly-active chronic colitis (Figure 4).

The colonic biopsy was not obtained due to friability of mucosa and the potential risk of perforation.

**Figure 5:**
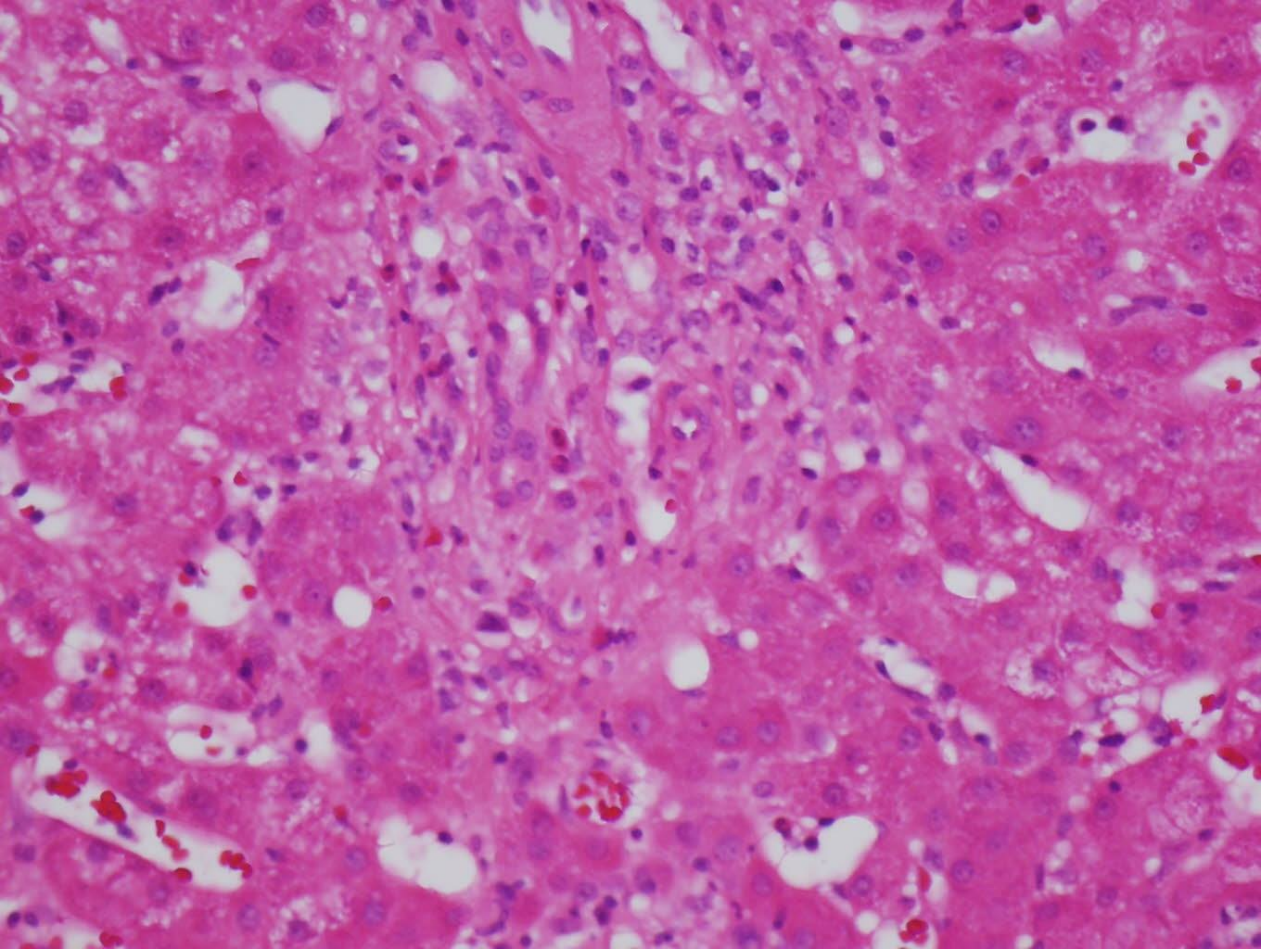
Liver Biopsy The biopsy image reveals significant eosinophilic infiltration in the portal triad. The surrounding periportal hepatocytes show mild steatosis.

On day 4 of admission, a repeat blood workup showed a white cell count of 23,700/mm^3^ with eosinophils 67% and an absolute total eosinophil count of 15,879/mm^3^. The liver function tests were markedly elevated. On hospital day 5, a liver biopsy was pursued to investigate the possibility of autoimmune hepatitis based on the laboratory abnormalities. The histopathologic analysis of the liver specimen revealed chronic active hepatitis with significant eosinophilic infiltration in the portal triad, but no interface activity or rosette formation was evident. The surrounding periportal hepatocytes showed mild steatosis (Figure 5).

**Figure 6:**
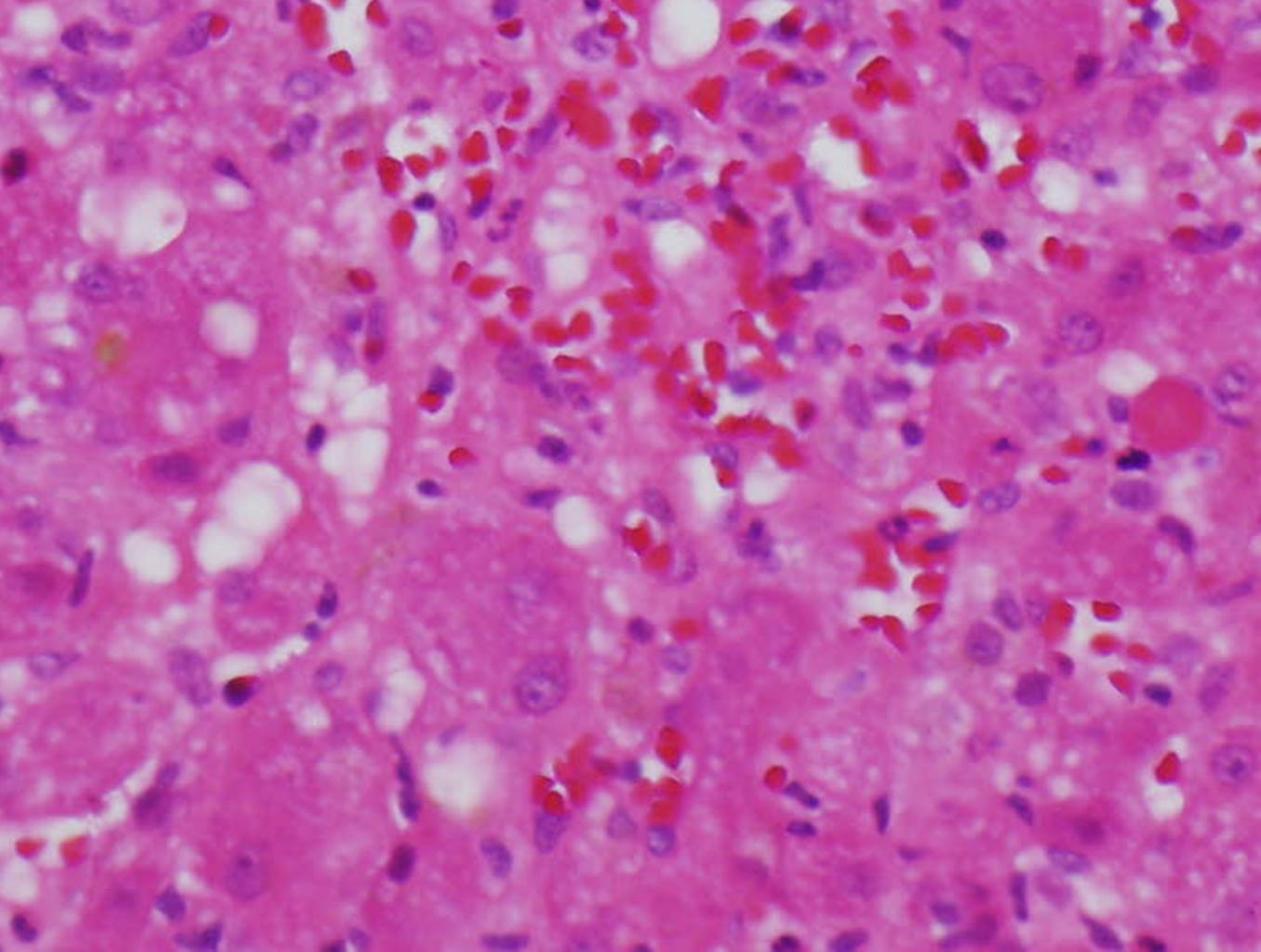
Liver Biopsy The high power view of the liver biopsy demonstrates infiltrating eosinophils with steatosis and mild cholestasis presenting as intrahepatocytic bile pigment.

On high power view of the liver biopsy, infiltrating eosinophils were found with mild cholestasis presenting as intrahepatocytic bile pigment (Figure 6).

Based on the calculator from the International Autoimmune Hepatitis Group, the patient’s clinical picture was not consistent with a definitive diagnosis of autoimmune hepatitis. Given the significant presence of eosinophils, the patient most likely had chronic hepatitis secondary to HES.

Eventually, the patient was diagnosed with idiopathic HES based on the persistent elevation in eosinophil count for more than six months, an otherwise negative workup as described above, and evidence of end-organ involvement of the liver, gallbladder, and colon. The patient was initiated on prednisone 60 mg/day. He showed a dramatic response to the prednisone therapy with normalization of eosinophil count within three weeks. The liver enzymes normalized after six weeks of therapy. Subsequently, the prednisone dose was gradually tapered. Since then, the patient has been disease free and continues to do well on regular follow-ups with serum troponin level measurements every three to six months and echocardiography and pulmonary function tests every six to 12 months.

## Discussion

Idiopathic HES is defined as a persistent absolute eosinophil count >1500/mm^3 ^for more than six months with evidence of end-organ dysfunction due to hypereosinophilia and exclusion of other primary or secondary causes of hypereosinophilia [4]. Patients with HES usually present with nonspecific symptoms or without symptoms [5]. In the majority of cases, it was shown that hematologic, cardiovascular, cutaneous, and neurologic systems have a relatively higher predilection to be affected in patients with HES [6].

As is clear from the definition of HES, it involves the hematologic system in all cases. Neutrophilia, anemia, thrombocytopenia, venous thrombosis, and splenomegaly may constitute additional symptoms in such cases [5-6]. Significant morbidity and mortality has been associated with HES cases with cardiovascular involvement [6]. The neurologic complications of HES include encephalopathy, peripheral neuropathy, and thromboembolic events of the central nervous system [6]. Cutaneous lesions seen in HES include urticaria and angioedema or erythematous pruritic papules and nodules with biopsies showing a range of findings including eosinophilic vasculitis or nonspecific inflammatory infiltration [6].

Gastrointestinal involvement in HES is well established. Eosinophilic gastritis, enterocolitis, or colitis may be present. Pancreatitis and sclerosing cholangitis occur rarely [6]. However, the initial clinical manifestations are not as well-defined and the majority of the data consists of case reports and case series. When the gastrointestinal tract is involved, patients may present with diarrhea, nausea, or abdominal cramps with subsequent biopsies showing eosinophilic infiltration and stool examination showing Charcot-Leyden crystals [5]. However, our patient initially presented with jaundice, and the sigmoidoscopy showed signs of chronic colitis. After the exclusion of potential etiologies, the symptoms in our patient were attributed to idiopathic HES.

Hepatic involvement in HES has been reported in up to one-third of patients and typically presents as hepatomegaly or mild abnormalities in liver chemistry studies [5]. The Budd-Chiari syndrome from hepatic vein obstruction may manifest following hepatic involvement in HES [6]. However, clinically relevant liver disease is less common, but isolated cases have been reported with patients presenting with hepatocellular damage due to chronic active hepatitis [7-8]. Hepatitis associated with HES has frequently been designated as chronic active hepatitis, eosinophilic hepatitis, and hepatitis associated with HES [8].

There are previous studies involving patients with HES who were initially suspected to have acute hepatitis, but were eventually diagnosed with chronic hepatitis based on additional biopsy findings after undergoing steroid therapy  [8]. In addition, Minola and Sonzogni reported a case of acute hepatitis in a patient with eosinophilic infiltration in the lung, which was finally proven to be chronic hepatitis [9]. Additional cases have been described in patients with hypereosinophilia and liver biopsies showing acute hepatitis, but in the setting of suspected IgG4-related disease [10]. Hence, as in the present case, HES may cause chronic liver injury.

## Conclusions

The present study highlights a unique case of a patient who meets the WHO diagnostic criteria for idiopathic HES with evidence of liver, gallbladder, and colon involvement. The patient had gastrointestinal involvement with no clinical evidence of the more commonly affected pulmonary, neurologic, or integumentary system dysfunction. Additionally, this patient had chronic active hepatitis related to the HES that may have otherwise been masked by the suspicion for autoimmune hepatitis based on serologic workup. Therefore, idiopathic HES may be considered among the differentials in cases with chronic active hepatitis, jaundice, and watery diarrhea.

## Appendices

The patient agreed to participate and was explained the nature and objectives of this study, and informed consent was formally obtained from the patient. No reference to the patient's identity was made at any stage during data analysis or in the report.
